# Overexpression of tousled-like kinase 2 predicts poor prognosis in HBV-related hepatocellular carcinoma patients after radical resection

**DOI:** 10.3389/fgene.2023.1326737

**Published:** 2024-01-26

**Authors:** Bang Liu, Ling-Ling Lu, Li Yu, Xuan Mei, Jia Liu, Jiao-Long Zheng, Xiao-Ling Zhou, Hai-Yan Lin, Xiu-Ling Zhu, Dong-Liang Li

**Affiliations:** ^1^ Fuzong Clinical Medical College of Fujian Medical University, Fuzhou, China; ^2^ Department of Hepatobiliary Disease, 900th Hospital of Joint Logistics Support Force, Fuzhou, China; ^3^ Department of Infectious Disease, Fujian Medical University Union Hospital, Fuzhou, China; ^4^ Department of Digestive Diseases, 900th Hospital of Joint Logistics Support Force, Fuzhou, China; ^5^ Department of Hepatobiliary Surgery, The Fifth Medical Center of Chinese PLA General Hospital, Beijing, China

**Keywords:** Tlk2, HBV-related HCC, prognostic, immune infiltration, enrichment analysis

## Abstract

**Background:** Tousled-like kinase 2 (TLK2) is integral to DNA repair, replication, and cell cycle regulation, crucial for maintaining genome stability and integrity. However, the expression and prognostic value of TLK2 in hepatitis B viral (HBV) -related hepatocellular carcinoma (HCC) remains unclear.

**Methods:** We examined TLK2 expression and prognostic implications in pan-cancer by using diverse databases. Subsequently, TLK2 expression in HBV-related HCC tissues and adjacent tissues was assessed using quantitative real-time PCR and immunohistochemistry. The prognostic value of TLK2 was assessed through ROC curves, time-dependent ROC curves, Cox regression, Kaplan-Meier curve, and decision curve analysis. Additionally, analyses of immune infiltration, protein-protein interactions, key molecules of tumor-related signaling pathways, molecular subtypes, and TLK2-associated differentially expressed genes (DEGs) were conducted, along with GO/KEGG and GSEA enrichment analyses.

**Results:** TLK2 expression was significantly higher in HCC tissues compared to adjacent tissues and correlated with gender, AFP levels, albumin-bilirubin (ALBI) grade, microvascular invasion (MVI), maximum tumor diameter, tumor number, and TNM stage. TLK2 overexpression emerged as an independent risk factor for overall survival (OS) and recurrence-free survival (RFS) in HBV-related HCC patients. An integrated OS nomogram model, incorporating TLK2, age, ALBI grade, MVI, and tumor number, displayed enhanced prognostic capability (C-index: 0.765, 95% CI: 0.732–0.798) in predicting OS and has a higher net benefit than the TNM stage. Moreover, TLK2 expression correlated closely with immune cell infiltration and key molecules of signaling pathways. Functional enrichment analyses highlighted significant associations with DNA duplex unwinding, double-strand break repair, DNA replication, cell cycle, E2F targets, G2M checkpoint, and MYC targets V1.

**Conclusion:** TLK2 is notably overexpressed in HBV-related HCC and emerges as a promising prognostic biomarker, necessitating further validation.

## 1 Introduction

Primary liver cancer, most of which is hepatocellular carcinoma (HCC), ranks sixth in global cancer incidence and third in mortality rates, contributing to 8.3% of cancer-related deaths (Sung et al., 2021). In China, up to 80% of HCC cases are related to HBV infection (Wang et al., 2014). Infection with HBV causes chronic hepatic inflammation, which leads to persistent hepatic injury and triggers liver fibrosis. The Hepatitis B virus integrates into the host genome, which causes chromosomal instability (CIN) and oncogene activation via insertion mutations and promotes hepatocarcinogenesis (Wang et al., 1990). Additionally, long-term expression of HBx protein and/or preS/S envelope proteins may contribute to the dysregulation of cell transcription and proliferation, resulting in hepatocarcinogenesis (Levrero and Zucman-Rossi, 2016). Reduction in intrahepatic covalently closed circular DNA (cccDNA) and HBV integration were observed in chronic hepatitis B (HBV) patients achieving functional cure (Gan et al., 2023). Even though the risk of HCC can be reduced by HBV antiviral therapy, it cannot be avoided completely (Hou et al., 2020).

About 66% of HCC patients are diagnosed at intermediate to advanced stages, often missing the window for radical resection (Park et al., 2015). Even among those eligible for surgery, nearly 70% experience metastasis and recurrence within 5 years post-surgery (Marrero et al., 2018). While advancements in diagnostics and therapeutics have been notable, HCC outcomes continue to present challenges. The predominant biomarker for HCC diagnosis and efficacy evaluation, α-fetoprotein (AFP), falls short for 30%–40% of HCC patients, particularly those with smaller tumors or at early stages, limiting its utility in diagnosis and prognosis (Farinati et al., 2006; Giannini et al., 2012). The Barcelona Clinic Liver Cancer (BCLC) staging system, too stringent in radical resection criteria, faces criticism due to marked clinical outcome disparities among patients of the same stage, especially in stage B (Zhong et al., 2014; Zhaohui et al., 2019). Hence, there’s an urgent need for an effective prognostic biomarker for HCC, accompanied by an exploration of the underlying mechanisms.

TLK2, a member of the serine-threonine kinase family, is integral to DNA replication and repair, virus latency, and cell cycle checkpoint control (Segura-Bayona and Stracker, 2019). Tousled-like kinases (TLKs) are pivotal for genome stability and normal development across species (Mortuza et al., 2018), with peak activity during the S-phase (Ehsan et al., 2004). They’re also targeted by DNA damage checkpoints (Groth et al., 2003) and impact anti-silencing factor 1 (ASF1), influencing chromatin structure (Silljé and Nigg, 2001; Klimovskaia et al., 2014). TLK2 mutations have been linked to neurological disorders, such as mental disabilities, behavior disorders, autism spectrum disorder (Lelieveld et al., 2016; Reijnders et al., 2018), and Alzheimer’s disease (Wang et al., 2010). Both genomic or epigenome instability and over-proliferation play key roles in cancer etiology (Segura-Bayona and Stracker, 2019). Chromosome instability is one of the earliest genetic changes in precancerous lesions. However, TLK2 overexpression is common in certain tumors, correlating with adverse clinical outcomes (Kim et al., 2016b; Lin et al., 2019; Wang et al., 2023). There are no studies have been reported to explore the effects and possible pathogenesis of TLK2 in HBV-related HCC. Utilizing clinical data and multiple public databases, we determine TLK2’s significant prognostic value in HCC and explore possible underlying molecular mechanisms via bioinformatics analysis.

## 2 Materials and methods

### 2.1 Data collection and definitions

TLK2 mRNA expression and relevant clinical data were obtained from various databases: the Cancer Genome Atlas (TCGA) database (https://portal.gdc.cancer.gov/) (Weinstein et al., 2013), Gene Expression Omnibus (GEO) database (https://www.ncbi.nlm.nih.gov/geo/) (Barrett et al., 2013), TIMER database (http://timer.comp-genomics.org/) (Li et al., 2017), and UALCAN database (http://ualcan.path.uab.edu) (Chandrashekar et al., 2017; Chandrashekar et al., 2022). The RNA sequencing data were calculated as transcripts per million (TPM) and normalized using the log_2_(TPM+1) transformation. Data processing and graph generation were conducted using R software (version 4.2.1) based on the Xiantao platform (https://www.xiantaozi.com/). Statistically significant differences were determined at a threshold of *p* < 0.05, and these differences were represented as follows: *p* > 0.05 (ns), *p* < 0.05 (*), *p* < 0.01 (**), and *p* < 0.001 (***).

We randomly enrolled a total of 240 patients who had undergone initial and curative resection for HBV-related HCC at 900th Hospital of Joint Logistics Support Force between April 2016 and December 2020. Among these patients, forty-eight pairs of HCC tissues and corresponding para-carcinoma tissues were collected. The inclusion and exclusion criteria were as follows: (1) Pathological confirmation of HCC; (2) HBV surface antigen (HBsAg) positive for at least 6 months and/or HBV-DNA positive; (3) R0 tumor resection; (4) complete clinical data (including preoperative imaging, laboratory examination, and postoperative pathology) and follow-up data; (5) absence of previous malignancies; and (6) no preoperative anticancer treatments. The follow-up deadline was 31 December 2022. The median follow-up time was 1,510 days [interquartile range (IQR): 1,377–1,647]. Over the follow-up duration, 131 patients (54.6%) experienced recurrence, and 65 patients (27.1%) succumbed. Approval from the Ethics Committee of the 900th Hospital of Joint Logistics Support Force was obtained, and informed consent was acquired from all participating patients. The interval between the surgical procedure and either death or recurrence was designated as OS and RFS, respectively. The ALBI score was calculated using the formula: log10 total bilirubin (μmol/L) × 0.66 - albumin (g/L) × 0.085. This score is divided into three categories: grade 1 (≤-2.60), grade 2 (between −2.60 and −1.39) and grade 3 (>-1.39) (Johnson et al., 2015).

### 2.2 Quantitative real-time PCR (qRT-PCR) and immunohistochemistry (IHC)

The qRT-PCR was performed to evaluate the expression of TLK2 in 48 paired samples by using SYBR Select Master Mix (4,472,908, Thermo Fisher) and an ABI 7900HT system. TLK2 primers: CTG​AAG​CAA​AGG​CGT​TTA​TTC​G (forward), GGA​TGT​GAG​GCA​ACA​AGT​AGG (reverse). β-actin primers: 5′-TGA CGTGGACATCCG CAAAG3' (forward), 5′-CTG​GAA​GGT​GGA​CAG​CGA​GG-3' (reverse). The PCR cycling conditions were as follows: 50°C for 2 min, 95°C for 2 min, then 95°C for 15 s, and 60°C for 1 min, 40 cycles. The qRT-PCR data were analyzed utilizing the 2^−ΔΔCT^ method.

Immunohistochemical staining was conducted on 240 HBV-related HCC cases. The retrieval of antigens was accomplished by utilizing Tris-EDTA buffer with a pH of 9.0. The anti-TLK2 antibody (13979-1-AP, Proteintech) was applied at a 1:150 dilution and incubated for 2 h at room temperature. Subsequently, the secondary antibody (Kit-9901, Fuzhou Maixin Biotech) was added and incubated at room temperature for 30 min. Next, color development was achieved by adding 3, 3′-diaminobenzidine (DAB). The TLK2 protein levels were semi-quantitatively assessed via immunohistochemistry. The proportion score was categorized as follows: scored 0 (0%–5% positive cells), scored 1 (5%–25% positive cells), scored 2 (25%–50% positive cells), scored 3 (50%–75% positive cells), scored 4 (75%–100% positive cells). The intensity of staining was assessed and categorized as: scored 0 (negative staining), scored 1 (weak staining), scored 2 (moderate staining), scored 3 (strong staining). IHC scores were determined by multiplying the proportion score by the staining intensity score. High TLK2 expression was classified as IHC scores ≥3, while low TLK2 expression was defined as IHC scores <3.

### 2.3 Correlation between TLK2 expression and clinicopathological features of HBV-related HCC patients

Using data from the 900th Hospital, we employed the Student’s t-test, Wilcoxon test, and Chi-squared test to compare clinicopathological features between the low and high TLK2 expression groups. The analysis of statistics was performed using the ‘stats’ package in the R software.

### 2.4 Prognostic assessment of TLK2

To evaluate the prognostic value of TLK2, we performed both univariate and multivariate Cox regression analyses to identify independent risk factors for OS and RFS. Stepwise multivariate Cox regression analysis, with backward elimination of significant risk factors (*p* < 0.1) from the univariable analysis, was performed. Independent risk factors for OS, including TLK2, age, ALBI grade, MVI, and tumor number, were integrated into an OS nomogram model to evaluate the outcome in patients with HBV-related HCC. The model’s performance was assessed utilizing the concordance index (C-index), calibration curve, time-dependent ROC curve, KM curve, and decision curve analysis (DCA). Data analysis and plotting were executed using the ‘survival’, ‘rms’, ‘survminer’, ‘timeROC’, ‘pROC’, ‘stdca.R’, and ‘ggplot2’ packages.

### 2.5 Association between TLK2 expression and immune infiltration

Tumor immune cell infiltration data are available in a previous article (Bindea et al., 2013). Single sample gene set enrichment analysis (ssGSEA) algorithm was utilized for investigating the correlation between TLK2 expression and the infiltration levels of 24 immune cell types using the GSVA package version 1.46.0. The above data were analyzed on the online platform of Xiantao. Five immune cell types with an absolute correlation coefficient value higher than 0.3, as determined by Spearman’s correlation test, were identified and visualized using scatter plots.

### 2.6 Construction of TLK2-associated protein-protein interaction (PPI) network

We obtained data for 50 interacting proteins of TLK2 from the STRING database (https://string-db.org, version 12.0), using the following parameters: required score set to medium confidence (0.400), and a size cutoff of no more than 50 interactors (Szklarczyk et al., 2023). The PPI network comprising these interacting proteins was visualized using Cytoscape (version 3.9.1) (Shannon et al., 2003). The Venn diagram was utilized to exhibit the three overlapping hub proteins of the results of the five algorithms. The relationships between TLK2 expression and the expression of genes corresponding to the three overlapping hub proteins were evaluated via scatterplots based on the GSE 121248 dataset. Then, we conducted Gene Ontology (GO) and Kyoto Encyclopedia of Genes and Genomes (KEGG) enrichment analyses for the 50 TLK2-associated proteins. These analyses were carried out utilizing the ‘VennDiagram’, ‘clusterProfiler’, and ‘ggplot2’ packages.

### 2.7 Correlation between TLK2 and several key molecules of tumor-related signaling pathways expression and molecular subtypes

Based on the TCGA LIHC dataset, we employed the Spearman correlation analysis was performed to assess the correlation between TLK2 expression and the expression of several key molecules of tumor-related signaling pathways, including catenin beta 1 (CTNNB1), AXIN1, phosphatase and tensin homolog (PTEN), tumor protein p53 (TP53), kelch like ECH associated protein 1 (KEAP1), cyclin dependent kinase inhibitor 2A (CDKN2A), cyclin D1 (CCND1), and FGF19. According to the information provided by a previous article (2017), HCC patients from the TCGA LIHC dataset were classified into three molecular subtypes (i.e., iCluster 1, iCluster 2, and iCluster 3). Games-Howell test and KM survival analysis were used to assess TLK2 expression differences and prognosis differences among molecular subtypes, respectively. The R packages ‘stats’, ‘car’, ‘survival’, ‘survminer’, and ‘ggplot2′ were utilized for performing data analysis and visualization.

### 2.8 TLK2 differential gene expression analysis

Differential gene expression analysis between the high and low TLK2 groups according to the median TLK2 expression in patients with HBV-related HCC was performed using the gene expression profile of GSE 121248. Differential gene expression data were visualized as a volcano plot. The differential gene expression threshold was adjusted *p* < 0.05 and |log2 fold change|>1. The top 10 hub genes among 46 DEGs were identified using five algorithms of the cytoHubba plugin in Cytoscape. The Venn diagram was utilized to exhibit the five overlapping hub genes of the results of the five algorithms. The relationships between TLK2 expression and the expression of five overlapping hub genes were evaluated via scatterplots. The above analyses were performed with ‘DESeq2’, ‘edgeR’, ‘VennDiagram’, and ‘ggplot2’ packages. The gene set enrichment analysis (GSEA) of DEGs was performed using ‘clusterProfiler’ packages. The ‘h.all.v7.5.1. symbols.gmt (Hallmarks)’ was selected as the reference gene set. Adjusted *p* < 0.05 and false discovery rate (FDR) < 0.25 were considered significantly different.

## 3 Results

### 3.1 Differential TLK2 expression and prognostic implications in pan-cancer analysis

**FIGURE 1:**
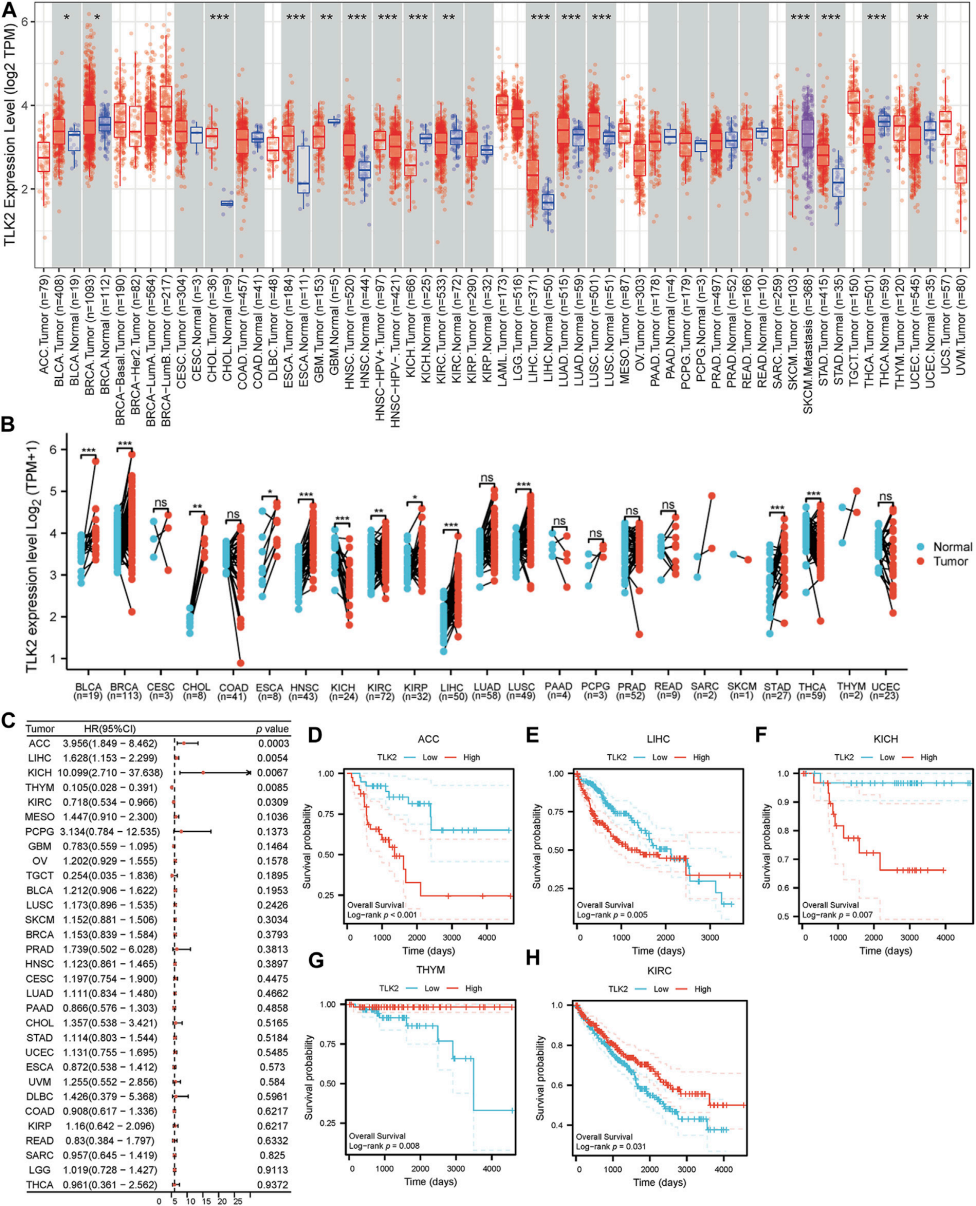
TLK2 mRNA expression and survival analysis in the pan-cancer. **(A)** The mRNA expression of TLK2 in pan-cancer analysis by TIMER database. **(B)** TLK2 mRNA expression in pan-cancer between tumor tissues and paired-adjacent normal tissues in TCGA. **(C)** The forest plot shows the association between TLK2 expression and OS in pan-cancer. KM survival curves revealed that high TLK2 expression correlates with poor OS in ACC **(D)**, LIHC **(E)** and KICH **(F)**, but longer OS in THYM **(G)** and KIRC **(H)**. (*p*-value: ns > 0.05; * <0.05; ** <0.01, *** <0.001).

Using the TIMER database, we observed varying TLK2 expression levels in different cancer types. TLK2 expression was upregulated in bladder urothelial carcinoma (BLCA), breast invasive carcinoma (BRCA), cholangiocarcinoma (CHOL), esophageal carcinoma (ESCA), head and neck squamous cell carcinoma (HNSC), LIHC, lung adenocarcinoma (LUAD), lung squamous cell carcinoma (LUSC), and stomach adenocarcinoma (STAD), while it was downregulated in glioblastoma multiforme (GBM), kidney chromophobe (KICH), kidney renal clear cell carcinoma (KIRC), thyroid carcinoma (THCA), and uterine corpus endometrial carcinoma (UCEC) (Figure 1A). Additionally, we assessed TLK2 expression in tumor tissues and matched-adjacent normal tissues using TCGA data. The results indicated TLK2 overexpression in BLCA, BRCA, CHOL, ESCA, HNSC, KIRC, kidney renal papillary cell carcinoma (KIRP), LIHC, LUSC, and STAD, but underexpression in KICH and THCA (Figure 1B). Forest plot of univariate COX analysis and KM analysis were used to assess the prognostic value of TLK2 in pan-cancer. TLK2 overexpression predicted poor OS in adrenocortical carcinoma (ACC) (*p* < 0.001), LIHC (*p* = 0.005), and KICH (*p* = 0.007), while indicating longer OS in thymoma (THYM) (*p* = 0.008) and KIRC (*p* = 0.031) (Figures 1C–H).

### 3.2 High TLK2 expression in HCC

**FIGURE 2:**
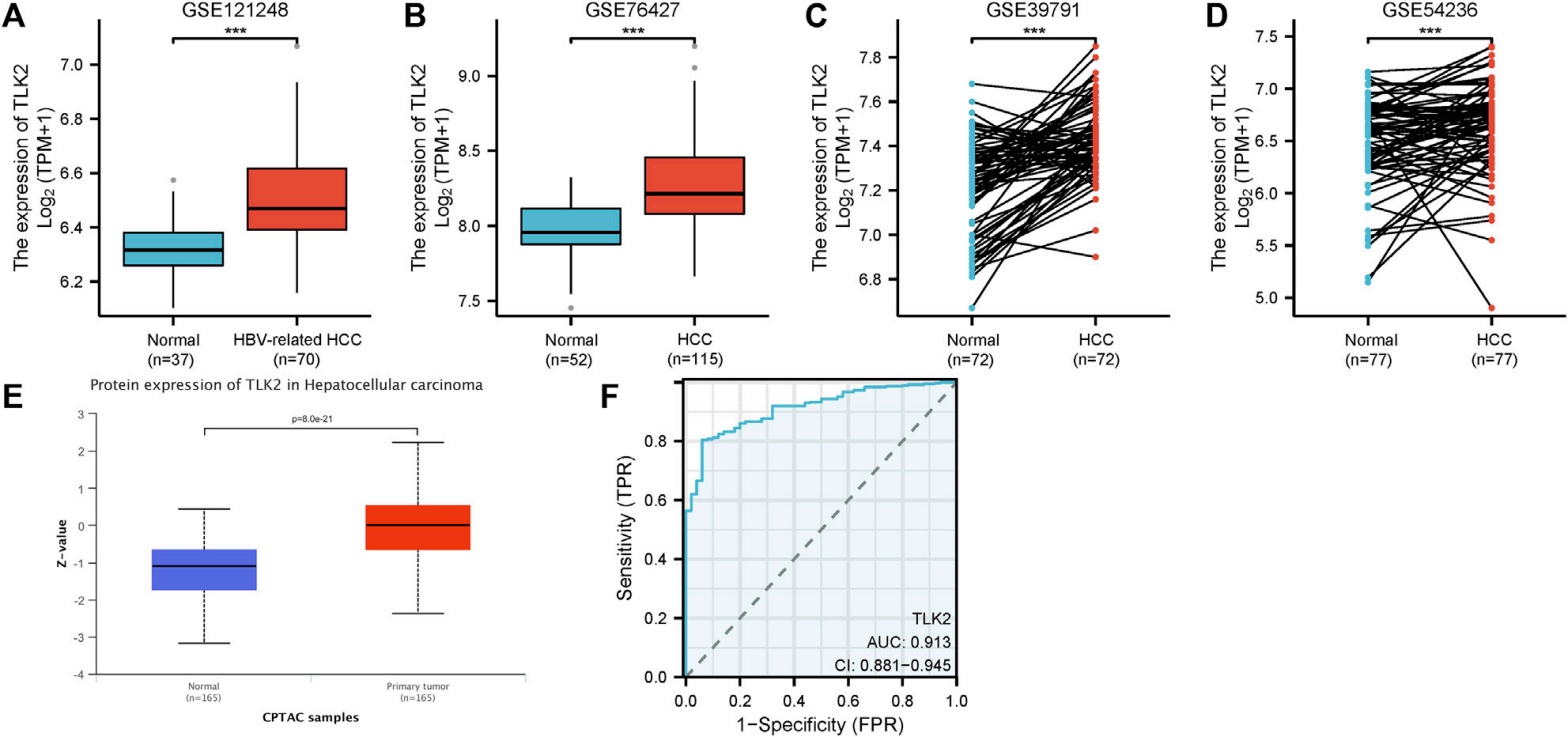
TLK2 expression of HCC patients in different databases. TLK2 expression levels were higher in the HCC group than in the normal group according to GEO databases [GSE 121248 **(A)**, GSE 76427 **(B)**, GSE 39791 **(C)**, and GSE 54236 **(D)**]. **(E)** TLK2 protein expression was significantly increased in HCC (obtained from the UALCAN website). **(F)** ROC curves of TLK2 discriminating LIHC tissues from normal tissues in TCGA.

The four GEO datasets, GSE 121248, GSE 76427, GSE 39791, and GSE 54236, showed that TLK2 mRNA expression levels are significantly higher in HCC tissue compared with normal liver tissue (*p* < 0.001 for all; Figures 2A–D). Moreover, based on the UALCAN database, TLK2 protein expression was notably elevated in HCC (Figure 2E). To assess the diagnostic precision of TLK2 expression, we conducted a ROC curve analysis, revealing a high discriminatory power between tumor and normal samples in LIHC (AUC = 0.913, 95%CI: 0.881 − 0.945) (Figure 2F).

### 3.3 Validation of TLK2’s high expression in HCC tissues from HBV-related HCC patients

**FIGURE 3:**
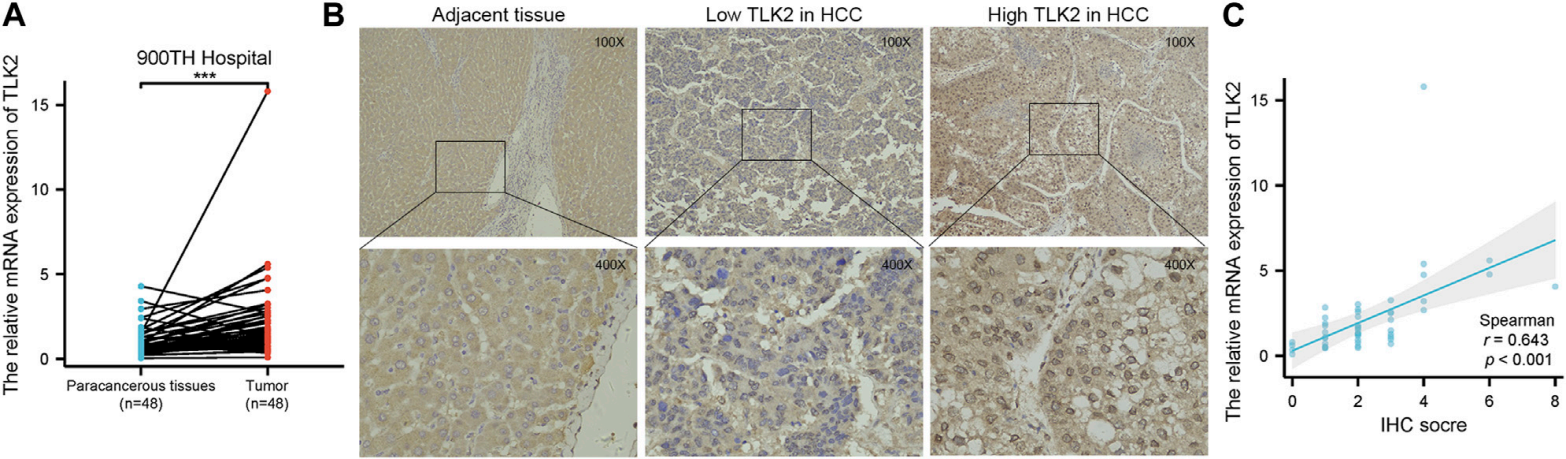
To validate the high expression of TLK2 in HBV-related HCC based on the samples of 900th hospital. **(A)** The mRNA expression of TLK2 was significantly higher in HCC tissues compared with paired adjacent normal tissues by qRT-PCR. **(B)** Representative IHC images of low (middle) and high (right) TLK2 expression in HCC (perinuclear staining), and negative staining in adjacent normal liver tissue (left). **(C)** Correlation between mRNA values and IHC scores for TLK2.

To verify the high expression of TLK2 in HCC tissues, we examined the relative mRNA expression of TLK2 in 48 pairs of HCC tissues and adjacent normal tissues using qRT-PCR. Additionally, TLK2 protein expression in 240 HCC tissues was evaluated through IHC. Results indicated that the expression of TLK2 mRNA in HCC tissues was significantly higher than in paired adjacent normal tissues (*p* < 0.001) (Figure 3A). TLK2 IHC demonstrates that perinuclear staining was increased in HCC tissues, whereas negative staining was in adjacent normal liver tissue (Figure 3B). Spearman correlation analysis indicates a significant positive correlation between the relative mRNA values and semiquantitative IHC scores for TLK2 (*r* = 0.643, *p* < 0.001; Figure 3C).

### 3.4 Correlation between TLK2 expression and clinicopathological features of patients with HBV-related HCC

Based on the IHC score of TLK2 expression, HBV-related HCC patients were divided into the high TLK2 expression group (n = 76) and the low TLK2 expression group (n = 164). Baseline clinicopathologic data, including gender, age, AFP, HBV-DNA, HBeAg, ALBI grade, cirrhosis, histologic grade, MVI, maximum tumor diameter, tumor number, and TNM stage, are detailed in Table 1. High TLK2 expression was strongly correlated with male gender (*p* = 0.028), higher levels of AFP (*p* = 0.033) and ALBI score (*p* < 0.001), worse MVI (*p* < 0.001), larger tumor diameter (*p* < 0.001), greater tumor number (*p* = 0.008) and more advanced TNM stage (*p* < 0.001).

**TABLE 1:** The clinical characteristics of HBV-related HCC patients in the TLK2 high and low expression group.

Characteristics	Low expression of TLK2 (n = 164)	High expression of TLK2 (n = 76)	*p* value
Gender, female/male	31/133 (18.9%/81.1%)	6/70 (7.9%/92.1%)	0.028
Age, years	53.55 ± 11.34	53.87 ± 11.52	0.840
<60/≥60	111/53 (67.7%/32.3%)	50/26 (65.8%/34.2%)	0.772
AFP, ng/ml	26.76 (4.96, 475.10)	51.33 (9.72, 1217.20)	0.033
<20	79 (48.2%)	31 (40.8%)	0.486
20 to 400	42 (25.6%)	20 (26.3%)	
≥400	43 (26.2%)	25 (32.9%)	
HBV-DNA, log IU/ml	2.98 (0, 4.58)	2.74 (0, 4.98)	0.736
negative/positive	71/93 (43.3%/56.7%)	35/41 (46.1%/53.9%)	0.689
HBeAg, negative/positive	134/30 (81.7%/18.3%)	60/16 (78.9%/21.1%)	0.613
ALBI score	−3.05 ± 0.31	−2.79 ± 0.35	<0.001
grade 1	152 (92.7%)	57 (75%)	<0.001
grade 2	12 (7.3%)	19 (25%)	
Cirrhosis, no/yes	80/84 (48.8%/51.2%)	36/40 (47.4%/52.6%)	0.839
Histologic grade			0.878
Ⅰ	24 (14.6%)	13 (17.1%)	
Ⅱ	110 (67.1%)	50 (65.8%)	
Ⅲ	30 (18.3%)	13 (17.1%)	
MVI, no/yes	133/31 (81.1%/18.9%)	47/29 (61.8%/38.2%)	0.001
Maximum tumor diameter, cm	3.55 (2.50, 5.43)	6.45 (3.65, 9.53)	<0.001
<2	25 (15.2%)	3 (3.9%)	<0.001
2 to 5	93 (56.7%)	26 (34.2%)	
≥5	46 (28%)	47 (61.8%)	
Tumor number, single/multiple	130/34 (79.3%/20.7%)	48/28 (63.2%/36.8%)	0.008
TNM stage (8th AJCC)			<0.001
ⅠA&ⅠB	105 (64%)	28 (36.8%)	
Ⅱ	42 (25.6%)	27 (35.5%)	
ⅢA	17 (10.4%)	21 (27.6%)	

Abbreviations: TLK2, tousled-like kinase 2; AFP, alpha-fetoprotein; HBV-DNA, hepatitis B virus DNA; HBeAg, hepatitis B e antigen; ALBI, albumin-bilirubin; MVI, microvascular invasion; TNM, TNM, classification of malignant tumors.

### 3.5 Prognostic value of TLK2 in HBV-related HCC

To identify independent risk factors for OS and RFS in patients with HBV-related HCC, both univariable and multivariable Cox regression analyses were conducted. The results revealed that TLK2 (HR: 1.863, 95%CI: 1.033–3.361; *p* = 0.039), age (HR: 0.478, 95%CI: 0.254–0.896; *p* = 0.021), ALBI grade (HR: 2.223, 95%CI: 1.158–4.267; *p* = 0.016), MVI (HR: 6.753, 95%CI: 2.870–15.890; *p* < 0.001) and tumor number (HR: 3.715, 95%CI: 1.201–11.485; *p* = 0.023) were the independent risk factors for predicting short OS, and TLK2 (HR: 1.538, 95%CI: 1.033–2.290; *p* = 0.034), ALBI grade (HR: 1.664, 95%CI: 1.021–2.711; *p* = 0.041) and MVI (HR: 2.688, 95%CI: 1.418–5.095; *p* = 0.002) were the independent risk factors for predicting poor RFS. The results of the Cox regression analyses are presented in Table 2. The KM curves of the aforementioned independent risk factors for OS and RFS are shown in Supplementary Figures S1, S2. When using the TCGA-LIHC database, TLK2 (HR: 1.626, 95%CI: 1.023–2.583; *p* < 0.05) remained an independent risk factor for OS (Supplementary Table S1).

**TABLE 2:** Cox regression analysis for OS and RFS in HBV-related HCC patients.

Characteristics	Total(N)	OS				RFS			
		Univariate analysis		Multivariate analysis		Univariate analysis		Multivariate analysis	
		Hazard ratio (95% CI)	*p* value	Hazard ratio (95% CI)	*p* value	Hazard ratio (95% CI)	*p* value	Hazard ratio (95% CI)	*p* value
Gender, female vs. male	37/203	2.460 (0.988 − 6.126)	0.053	1.821 (0.712 − 4.656)	0.211	1.840 (1.056 − 3.205)	0.031	1.569 (0.886 − 2.778)	0.122
Age, <60 vs. ≥60	161/79	0.478 (0.265 − 0.865)	0.015	0.478 (0.254 − 0.896)	0.021	0.697 (0.478 − 1.018)	0.062	0.797 (0.530 − 1.199)	0.277
AFP, ng/ml									
<20	110	Reference				Reference			
20 to 400	62	1.675 (0.927 − 3.028)	0.088			1.177 (0.765 − 1.812)	0.459		
≥400	68	1.517 (0.838 − 2.748)	0.169			1.506 (1.007 − 2.252)	0.046		
HBV-DNA, negative vs. positive	106/134	1.790 (1.064 − 3.012)	0.028	1.719 (0.989 − 2.989)	0.055	1.394 (0.979 − 1.984)	0.065	1.214 (0.839 − 1.756)	0.304
HBeAg, negative vs. positive	194/46	1.272 (0.714 − 2.265)	0.414			1.224 (0.810 − 1.850)	0.337		
ALBI grade, grade 1 vs. grade 2	209/31	2.470 (1.385 − 4.404)	0.002	2.223 (1.158 − 4.267)	0.016	1.889 (1.203 − 2.967)	0.006	1.664 (1.021 − 2.711)	0.041
Cirrhosis, no vs. yes	116/124	1.744 (1.053 − 2.886)	0.031	1.689 (0.980 − 2.913)	0.059	1.453 (1.027 − 2.055)	0.035	1.454 (0.995 − 2.125)	0.053
Histologic grade									
Ⅰ	37	Reference		Reference		Reference		Reference	
Ⅱ	160	1.673 (0.711 − 3.935)	0.239	1.506 (0.605 − 3.749)	0.379	1.229 (0.729 − 2.072)	0.439	1.076 (0.625 − 1.852)	0.793
Ⅲ	43	2.949 (1.162 − 7.483)	0.023	1.558 (0.582 − 4.171)	0.378	2.204 (1.210 − 4.015)	0.010	1.658 (0.892 −3.081)	0.110
MVI, no vs. yes	180/60	3.315 (2.033 − 5.404)	<0.001	6.753 (2.870 − 15.890)	<0.001	2.788 (1.936 − 4.015)	<0.001	2.688 (1.418 − 5.095)	0.002
Maximum tumor diameter, cm	240								
<2	28	Reference		Reference		Reference		Reference	
2 to 5	119	3.204 (0.759 − 13.529)	0.113	3.476 (0.759 − 15.922)	0.109	1.971 (0.939 − 4.136)	0.073	1.795 (0.829 − 3.888)	0.138
≥5	93	7.512 (1.811 − 31.159)	0.005	3.617 (0.693 − 18.887)	0.127	4.684 (2.239 − 9.795)	<0.001	2.286 (0.955 − 5.469)	0.063
Tumor number, single vs. multiple	178/62	2.430 (1.483 − 3.982)	<0.001	3.715 (1.201 − 11.485)	0.023	1.961 (1.366 − 2.816)	<0.001	1.078 (0.465 − 2.500)	0.862
TNM Stage (8th AJCC)									
ⅠA&ⅠB	133	Reference		Reference		Reference		Reference	
Ⅱ	69	3.086 (1.707 − 5.579)	<0.001	0.409 (0.136 − 1.226)	0.110	2.376 (1.593 − 3.545)	<0.001	0.964 (0.442 − 2.100)	0.926
ⅢA	38	4.771 (2.545 − 8.944)	<0.001	0.507 (0.093 − 2.754)	0.431	4.130 (2.662 − 6.406)	<0.001	1.807 (0.555 − 5.888)	0.326
TLK2, low vs. high	164/76	3.202 (1.964 − 5.220)	<0.001	1.863 (1.033 − 3.361)	0.039	2.357 (1.661 − 3.344)	<0.001	1.538 (1.033 − 2.290)	0.034

Abbreviations: OS, overall survival; RFS, recurrence-free survival; AFP, alpha-fetoprotein; HBV-DNA, hepatitis B virus DNA; HBeAg, hepatitis B e antigen; ALBI, albumin-bilirubin; MVI, microvascular invasion; TNM, TNM, classification of malignant tumors; TLK2, tousled-like kinase 2.

### 3.6 Construction of a nomogram for the prediction of OS in patients with HBV-related HCC

**FIGURE 4:**
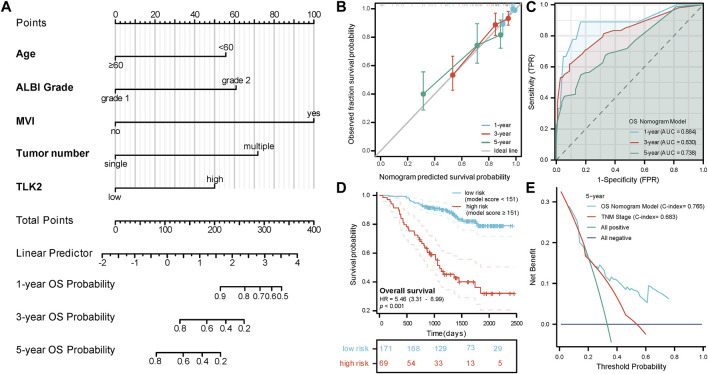
The prognostic value of TLK2 in HBV-related HCC. **(A)** Nomogram model construction based on five independent risk factors (including, age, ALBI grade, MVI, tumor number and TLK2) to predict 1-, 3-, and 5-year OS. **(B)** The calibration plot showed good consistency between observed and predicted OS probability. **(C)** Time-dependent ROC analysis of OS nomogram model for 1-, 3- and 5-year OS. **(D)** The KM curve for the high-risk and low-risk groups. **(E)** DCA curve of the OS nomogram model and the TNM stage for 5-year OS.

A nomogram model for predicting OS in patients with HBV-related HCC was created using the five independent risk factors (Figure 4A), and the model’s C-index was 0.765 (95%CI: 0.732–0.798), indicating a relatively good predictive capability. Calibration curves demonstrated good agreement between predicted and observed OS probabilities at 1-, 3-, and 5-year time points (Figure 4B). The time-dependent ROC curve of this model showed that the AUCs for 1-, 3-, and 5-year OS were 0.884, 0.830, and 0.738, respectively (Figure 4C). An optimal cut-off value for the total score of this model was determined through a 5-year time-dependent ROC curve analysis. Patients were categorized into low-risk (total score <151) and high-risk (total score ≥151) groups according to the total score obtained from the model. Prognosis varied significantly between the low-risk and high-risk groups (HR: 5.46, 95%CI: 3.31–8.99; *p* < 0.001) (Figure 4D). The low-risk group had 26 cases (15.2%) of death with a median OS of not reached, while the high-risk group had 39 cases (56.5%) of death with a median OS of 1,099 days (95% CI: 919 - not reached), respectively. DCA curves revealed that the OS nomogram model had a greater net benefit than the TNM stage (C-index: 0.683, 95% CI:0.651–0.715; *p* < 0.001) at 5 years (Figure 4E). These results showed that our model had well-discriminating ability and potential clinical benefits.

### 3.7 Correlation between TLK2 expression and immune cell infiltration

**FIGURE 5:**
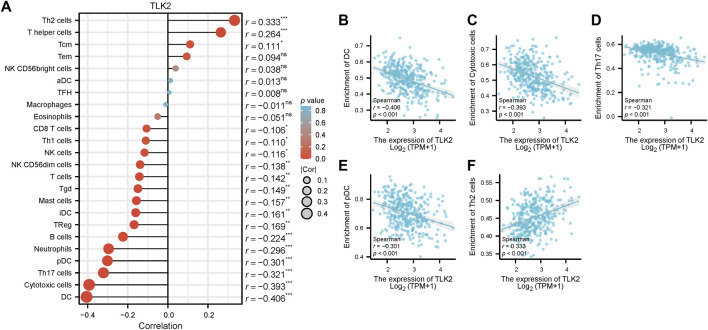
Correlation between TLK2 expression and immune cell infiltration. **(A)** The lollipop plot illustrated that TLK2 was correlated with 18 infiltrated immune cells. Scatter plots of TLK2 expression and the infiltration of DC **(B)**, cytotoxic cells **(C)**, Th17 cells **(D)**, pDC **(E)**, and Th2 cells **(F)** (Spearman correlation: |r| > 0.3).

Spearman’s correlation test was used to analyze the relationship between TLK2 expression and the levels of 24 immune cell types. Eighteen infiltrated immune cells exhibited significant correlations with TLK2 expression (Figure 5A), where significant negative associations with dendritic cells (DC), cytotoxic cells, Th17 cells, plasmacytoid DC (pDC), neutrophils, B cells, regulatory T cells (Treg), immature DC (iDC), mast cells, gamma-delta T cells (Tgd), T cells, natural killer (NK) CD56dim cells, NK cells, Th1 cells, and CD8 T cells; while positive correlations with Th2 cells, T helper cells and central memory T cells (Tcm). Among them, the correlation coefficient (absolute value of) higher than 0.3 were DC (*r* = −0.406, *p* < 0.001), cytotoxic cells (*r* = −0.393, *p* < 0.001), Th17 cells (*r* = −0.321, *p* < 0.001), pDC (*r* = −0.301, *p* < 0.001), and Th2 cells (*r* = 0.333, *p* < 0.001), which were demonstrated in scatter plots (Figures 5B–F). These findings suggest that TLK2 expression levels may influence the infiltration of certain immune cell types in HCC, underscoring the potential role of TLK2 in immune responses within the tumor microenvironment.

### 3.8 Protein-protein interaction analysis

**FIGURE 6:**
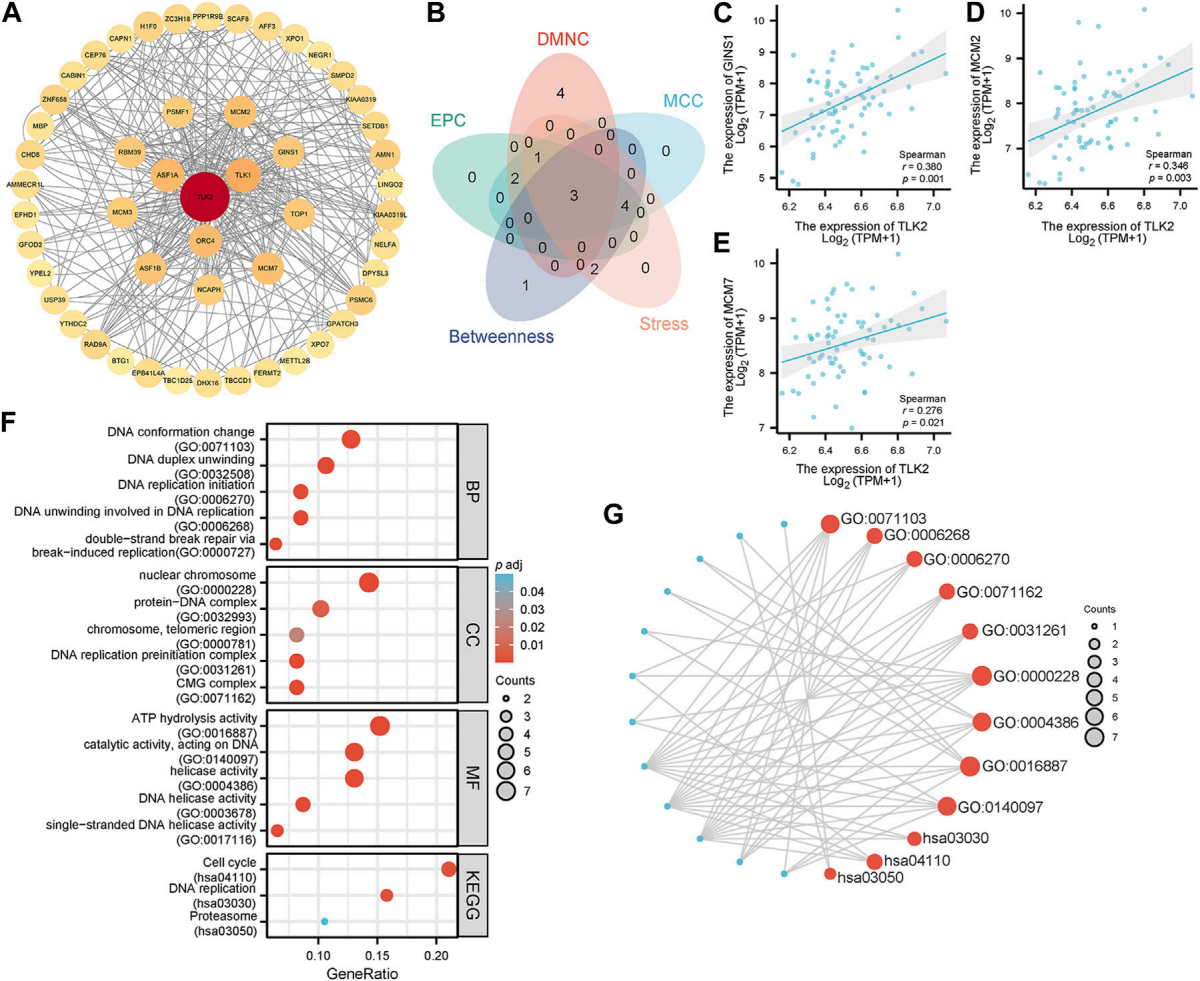
Protein-protein interaction (PPI) network, hub proteins, and GO/KEGG analysis of 50 interacting proteins of TLK2. **(A)** The PPI network of 50 interacting proteins of TLK2 was generated using the STRING database and Cytoscape (version 3.9.1). **(B)** Venn diagram of three overlapping hub proteins using five algorithms of the cytoHubba plugin in Cytoscape. Scatter plots of TLK2 expression and the expression of GINS1 **(C)**, MCM2 **(D)**, and MCM7 **(E). (F)** GO/KEGG pathway enrichment analysis for the interacting proteins of TLK2. **(G)** Visual network of GO/KEGG analysis.

Utilizing the STRING database, the study identified 50 interacting proteins of TLK2, which were then visualized using Cytoscape software (Figure 6A). We identified the top 10 hub proteins of 50 TLK2-associated proteins using five algorithms of the cytoHubba plugin in Cytoscape (Supplementary Table S2), with three overlapped hub proteins verified by a Venn diagram (Figure 6B), including Go-Ichi-Ni-San complex subunit 1 (GINS1), minichromosome Maintenance Complex Component 2 (MCM2), and MCM7. Scatter plots showed that the expression of genes corresponding to the three overlapping hub proteins significantly positively correlated with TLK2 expression (Figures 6C–E). GO analysis of these interacting proteins indicated that enriched biological process (BP) mainly including ‘DNA conformation change’, ‘DNA duplex unwinding’, ‘DNA replication initiation’, ‘DNA unwinding involved in DNA replication’, and ‘double-strand break repair via break-induced replication’; enriched cellular component (CC) primarily involved in ‘nuclear chromosome’, ‘protein-DNA complex’, ‘chromosome, telomeric region’, ‘DNA replication preinitiation complex’, and ‘CMG complex’; enriched molecular function (MF) mainly containing ‘ATP hydrolysis activity’, ‘catalytic activity, acting on DNA’, ‘helicase activity’, ‘DNA helicase activity’, and ‘single-stranded DNA helicase activity’. KEGG pathway analysis demonstrated significant enrichment in ‘cell cycle’, ‘DNA replication’, and ‘proteasome’ pathways (Figures 6F, G).

### 3.9 Correlation between TLK2 and several key molecules of tumor-related signaling pathways expression and molecular subtypes

**FIGURE 7:**
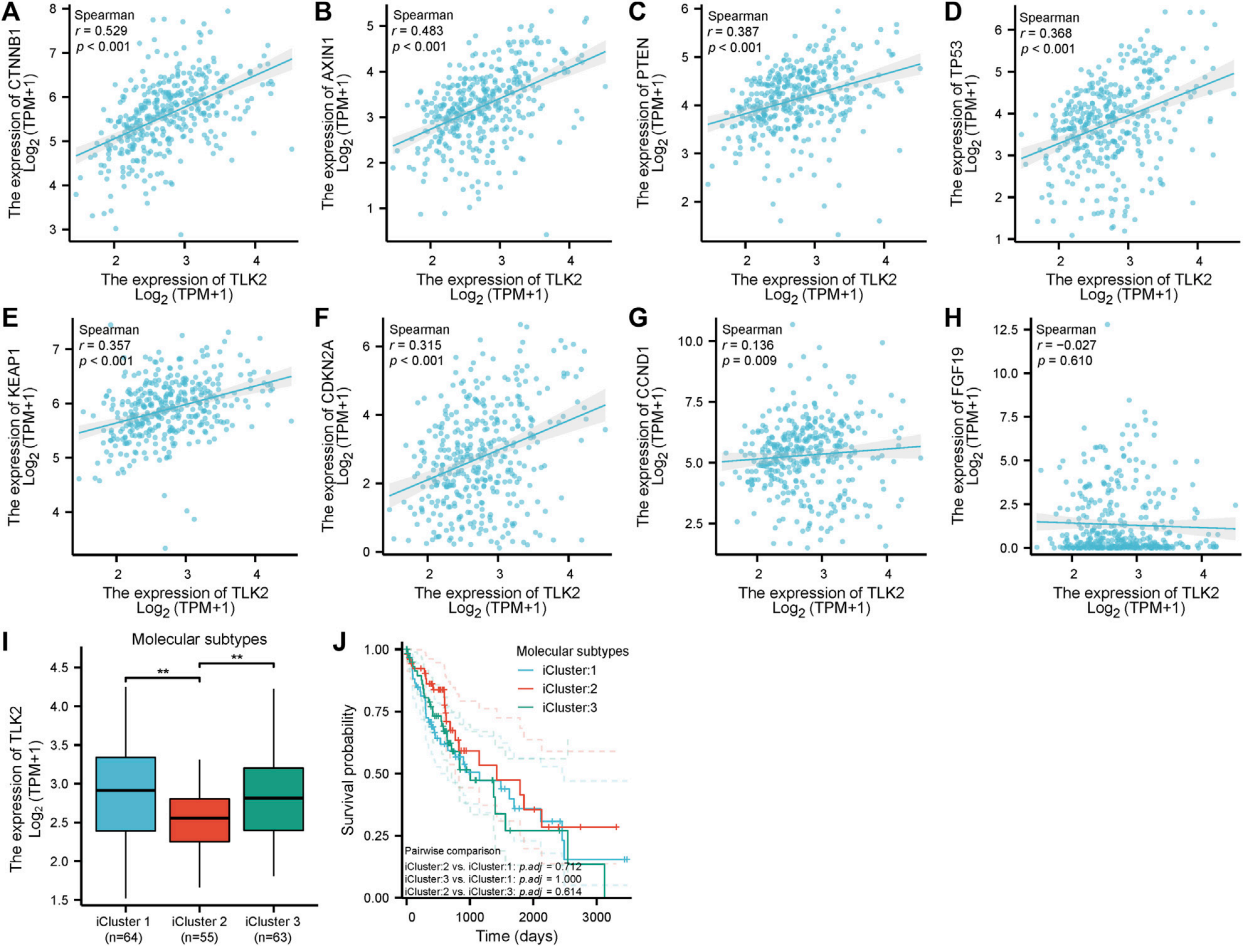
The correlation between TLK2 and several key molecules of tumor-related signaling pathways expression and molecular subtypes. Scatter plots of TLK2 expression and the expression of CTNNB1 **(A)**, AXIN1 **(B)**, PTEN **(C)**, TP53 **(D)**, KEAP1 **(E)**, CDKN2A **(F)**, CCND1 **(G)**, and FGF19 **(H)**. The mRNA expression of TLK2 **(I)** and KM survival analysis **(J)** in different HCC molecular subtypes.

Scatter plots showed that CTNNB1, AXIN1, PTEN, TP53, KEAP1, CDKN2A, and CCND1 were significantly positively correlated with TLK2 (*p* < 0.05 for all; Figures 7A–G). The results suggest that TLK2 expression might affect some tumor-related signaling pathways. The TLK2 expression levels in iCluster 1 and iCluster 3 were significantly higher than those in iCluster 2 (Figure 7I). However, no significant differences in KM survival analysis were observed among the three molecular subtypes (Figure 7J).

### 3.10 Differential gene expression analysis in patients with HBV-related HCC

**FIGURE 8:**
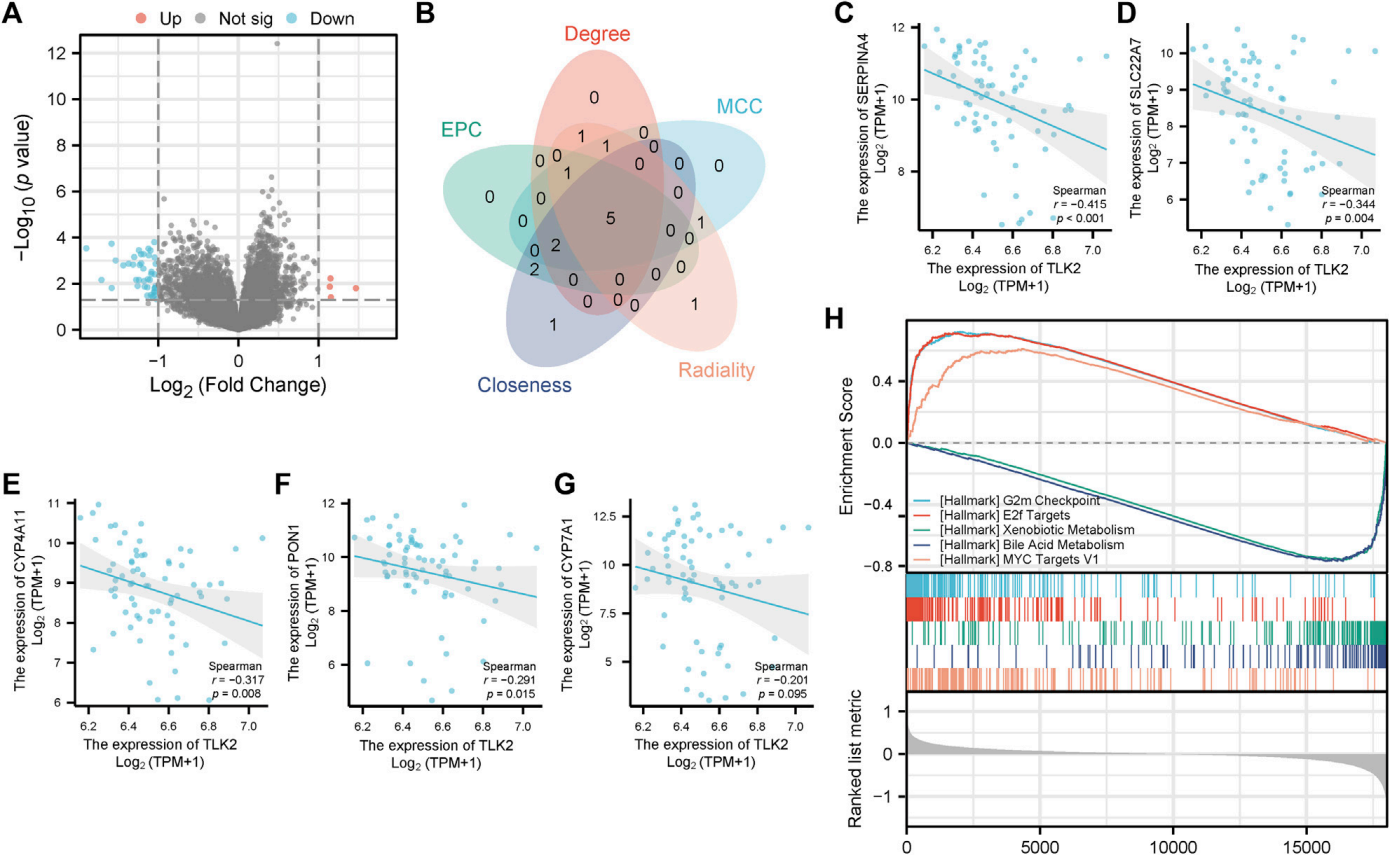
Differential gene expression analysis between the high and low TLK2 groups in patients with HBV-related HCC according to GSE 121248. **(A)** Volcano plot of the distribution of DEGs. **(B)** Venn diagram of five overlapping hub genes using five algorithms of the cytoHubba plugin in Cytoscape. Scatter plots of TLK2 expression and the expression of SERPINA4 **(C)**, SLC22A7 **(D)**, CYP4A11 **(E)**, PON1 **(F)**, and CYP7A1 **(G)**. **(H)** GSEA enrichment plots of the top five enriched gene sets using HALLMARK as a reference gene set.

The DEGs between TLK2-high and -low groups were visualized using a volcano plot (Figure 8A) based on the gene expression profile of GSE 121248. With a threshold of adjusted *p* < 0.05 and |log2 fold change|>1, a total of 46 DEGs were identified, including 4 upregulated genes and 42 downregulated genes. We identified the top 10 hub genes of DEGs using five algorithms of the cytoHubba plugin in Cytoscape (Supplementary Table S3), with five overlapped hub genes verified by a Venn diagram (Figure 8B), including SERPINA4, SLC22A7, CYP4A11, PON1, and CYP7A1. Scatter plots showed that SERPINA4, SLC22A7, CYP4A11, and PON1 were significantly negatively correlated with TLK2 (Figures 8C–F). A total of 32 gene sets were identified in the GSEA when using HALLMARK as a reference gene set, and the details of the top ten enriched gene sets are shown in Supplementary Table S4. The top five enriched gene sets included ‘G2M checkpoint’, ‘E2F targets’, ‘Xenobiotic metabolism’, ‘Bile acid metabolism’, and ‘MYC targets v1’ (Figure 8H).

## 4 Discussion

TLK2 selectively targets and phosphorylates ASF1, a histone H3/H4 chaperone, through client mimicry, which facilitates histone transfer to downstream histone chaperones for replication-coupled and chromatin assembly and fulfills the nucleosome demand for DNA replication (Segura-Bayona and Stracker, 2019; Simon et al., 2022). Inactivation of TLKs results in DNA replication fork pause, accumulation of single-stranded DNA (ssDNA), and cell cycle arrest in the G1 phase (Lee et al., 2018). Severe proliferative and mitosis defects and even apoptosis had been observed in *Drosophila* and *Caenorhabditis elegans* with the deactivation of TLKs (Carrera et al., 2003; Han et al., 2005). To cope with DNA damage, the body maintains the integrity of the genome through cell cycle checkpoints to identify breaks, repair, or withdraw from the cell cycle in time (Shaltiel et al., 2015). TLK2 is one of the key regulatory factors of DNA recovery after damage, which can restore DNA damage-induced G2 arrest (Bruinsma et al., 2016). TLK2 is not only required for normal development and maintenance of genome stability but is also closely related to the tumor. In luminal breast cancer, TLK2 overexpression interfered with the Chk1/2-induced G2/M DNA damage checkpoint signaling, which leads to prolongation of the DNA repair process and has an adverse effect on chromosome stability, thus inducing carcinogenesis (Kim et al., 2016a). TLK2 enhances the aggressiveness of breast cancer through the activation of the EGFR/SRC/FAK signaling pathway. However, inhibiting TLK2 leads to the downregulation of ERα, BCL2, and SKP2, causing cell cycle arrest in the G1/S phase and ultimately triggering apoptosis, which can help improve the prognosis of patients with breast cancer (Kim et al., 2016b). In glioblastoma, TLK2 overexpression correlates with poor clinical outcomes and drives cell proliferation, migration, invasion, and epithelial-mesenchymal transition through the SRC signaling pathway, while silencing TLK2 has opposite effects (Lin et al., 2019). Overexpressed TLK2 binds to ATF4 to enhance asparagine synthetase (ASNA) transcription level and prevent ubiquitination of ASNA, thus promoting the progression of gastric cancer (GC) by reprogramming amino acid metabolism. Inhibiting TLK2 activity by the mTOR/ASNS axis will result in a suppression of GC cell proliferation and invasion (Wang et al., 2023). These studies showed TLK2 may serve as a potential biomarker for poor prognosis in certain cancers, while its inhibition demonstrates potential for cancer therapy.

Here, we found that TLK2 mRNA is abnormally expressed in pan-cancer, with notable implications for prognosis in specific tumors, particularly in LIHC. This suggests that TLK2 might play a role as an oncogene in HCC and hold significant prognostic value. The molecular mechanism of TLK2 upregulation in HBV-related HCC is not clear. Existing studies have shown that CIN is often observed in HBV-related HCC, which is associated with insertional mutations and the activation of cancer driver genes due to HBV DNA randomly integration into the host genome (Guichard et al., 2012; Seeger and Mason, 2015; Péneau et al., 2021). Highly genome-unstable tumors tended to overexpress TLK2 to achieve immune escape through suppressing cGAS-STRING-TBK1-mediated innate immune response (Segura-Bayona et al., 2020). TLK2 overexpression may, in turn, further aggravate the risk of CIN (Kim et al., 2016a). Our research then zoomed in on HCC, where we confirmed elevated TLK2 expression in HCC tissues, utilizing multiple databases for validation. ROC curve analysis showed that TLK2 had high accuracy in discriminating tumor tissues from normal samples in LIHC (AUC = 0.913). However, there is no study on the relationship between TLK2 expression and HBV-related HCC patients’ prognosis. We investigated the role of TLK2 in HBV-related HCC by characterizing its expression using qRT-PCR and IHC. TLK2 mRNA expression in HCC tissues from HBV-related HCC patients was significantly higher than in paired adjacent normal tissues (*p* < 0.001). TLK2 overexpression means a higher level of AFP and ALBI score, worse MVI grade and TNM stage, larger tumor diameter, and greater tumor number in HBV-related HCC patients.

Multivariate Cox regression analysis revealed that TLK2 overexpression stands as an independent risk factor for predicting both unfavorable OS and RFS. Essentially identical result was observed in the TCGA-LIHC database, which verified the prognostic value of TLK2 again. Additionally, age, ALBI grade, MVI, and tumor number were all identified as independent risk factors for OS, and ALBI grade and MVI emerged as independent risk factors for RFS, which was consistent with that in earlier reports (Lim et al., 2011; Toyoda et al., 2016; Chan et al., 2018). However, HBV-DNA and HBeAg were not independent risk factors for OS and RFS, possibly due to the administration of anti-HBV treatment to all participants in our study. Some scholars have pointed out that antiviral treatment will improve the negative impact of elevated baseline HBV loads on the prognosis of HBV-related HCC patients (Hu et al., 2022). Interestingly, our findings indicated that younger patients (age<60) exhibited poorer OS compared to older patients (age≥60), which is contrary to many existing studies. Some reports indicate that younger patients often with larger tumor sizes, worse tumor stage, and more aggressive phenotype than older patients, which may account for this result (Furuta et al., 1990; Kim et al., 2006; Heo et al., 2014). Then, the five independent risk factors for OS were integrated into the OS nomogram model for evaluating prognosis in HBV-related HCC patients (Figure 4A). According to the total score derived from this model, patients were stratified into low- and high-risk groups and the high-risk group had a poorer prognosis than the low-risk group (HR: 5.46; 95%CI: 3.31–8.99). The model has better clinical benefit than the TNM stage (C-index = 0.765 vs C-index = 0.683, *p* < 0.001), which helps clinicians in assessing patient prognosis and identifying high-risk individuals among HBV-related HCC patients.

The tumor microenvironment (TME) has emerged as a critical factor in HCC development and has garnered significant attention in recent years (Chew et al., 2017). It represents a complex network of immunoregulation involving various immune cell types (Gajewski et al., 2013). The composition and status of tumor-infiltrating lymphocytes (TILs) within the TME have implications for immune therapy efficacy and HCC prognosis (Wada et al., 1998). Our results demonstrate that TLK2 expression is related to the infiltration levels of 18 immune cells, most notably negative correlations with DC, cytotoxic cells, Th17, and pDC while correlating positively with Th2 cells. DCs, as antigen-presenting cells, play an important role in the regulation of immune response and present tumor antigens to naive T cells, which can activate and induce naïve T cells to differentiate into cytotoxic T lymphocytes (CTLs) to kill tumor cells (Wierecky et al., 2006). However, compromised DC function is often observed in HBV-related HCC patients (Kakumu et al., 2000). The tumor microenvironment inhibits DC maturation and leads to DCs with an immunosuppressive phenotype (Motta and Rumjanek, 2016). HCC inhibits CTLs via recruiting immunosuppressive DC cells, which contribute to an immunosuppressive microenvironment in HCC and help the tumor evade immune surveillance (Villalba et al., 2013). DCs promoted naive T cells to differentiate into Th17 by IL-23 secretion (Blanco et al., 2008). Th17 cells are a pro-inflammatory T cell subset that can promote inflammatory reactions by secreting several inflammatory factors, including IL-17 (Littman and Rudensky, 2010). Th17/Treg balance is frequently dysregulated in CHB and HCC, which is associated with poor patient prognosis (Zhang et al., 2019). During the progression of CHB toward HCC, the density of liver-infiltrated Th17 cells decreased and that of Treg cells increased, gradually (Zhang et al., 2019). Th2 cells secrete IL-4 and IL-10 to mediate humoral immune responses, which leads to immune suppression and promotes tumor growth and metastasis (Lee et al., 2019). Increased Th2 cell numbers have been observed in the peripheral blood and HCC tissue of HCC patients and are associated with poor prognosis (Foerster et al., 2018; Sun et al., 2023). Based on the above results, we hypothesized that TLK2 has a significant impact on the HCC microenvironment by reducing DCs, pDCs, CTLs, and Th17 cell infiltration but increasing Th2 cell infiltration.

Using the CytoHubba plugin of Cytoscape software, 3 overlapping hub proteins, namely, GINS1, MCM2, and MCM7, were identified from the PPI network of 50 TLK2-associated proteins. The expression of genes corresponding to the above hub proteins significantly positively correlated with TLK2 expression, which was validated in an HBV-related HCC cohort of GSE 121248. GINS1 is essential for the establishment of DNA replication forks and the initiation of DNA replication and contributes to promoting hepatocellular cancer cell stemness and enhancing Sorafenib resistance (Kamada et al., 2007; Li et al., 2021). MCM2 and MCM7 are important components of the MCM2-7 complex and cooperate with GINS and CDC45 to promote the assembly of the CDC45-MCM2-7-GINS (CMG) helicase, a critical event in the initiation of DNA replication (Costa and Diffley, 2022). Previous studies showed that MCM2 and MCM7 promote hepatocellular carcinoma cell stemness and tumor progression via hippo signaling and cyclin D1-dependent signaling, respectively, and overexpression of them correlates with poor prognosis in HCC (Zhou et al., 2012; Qu et al., 2017; Tang et al., 2022; Zhou et al., 2022). GO analysis showed that the functions of TLK2 were mainly enriched in DNA replication initiation, DNA duplex unwinding, double-strand break repair, DNA helicase activity, and so on. KEGG pathway enrichment analysis demonstrated that TLK2 mainly participated in the cell cycle and DNA replication. Our findings are consistent with existing literature, highlighting TLK2’s significant involvement in DNA replication and repair, cell cycle-regulated, and chromosome segregation (Silljé and Nigg, 2001; Groth et al., 2003; Krause et al., 2003; Kim et al., 2016b; Mortuza et al., 2018). Additionally, we found that TLK2 expression was significantly positively associated with several key molecules of tumor-related signaling pathways (i.e., cell cycle control, WNT/beta-catenin signaling, AKT/m-TOR/MAPK signaling, and oxidative stress) in the TCGA LIHC dataset. We therefore speculate that TLK2 cooperates with multiple molecules and signaling pathways in promoting HCC oncogenesis.

Then, we performed differential gene expression analysis in a cohort of HBV-related HCC patients from GSE 121248, and then four overlapped hub genes, SERPINA4, SLC22A7, CYP4A11, and PON1, were identified, which were significantly negatively correlated with TLK2. The above four hub genes were reported to be associated with the prognosis of patients with HCC (Yasui et al., 2014; Wu et al., 2019; Eun et al., 2019; Ruan et al., 2023). Therefore, we hypothesized that TLK2 and hub genes may have a reciprocal influence and consequently promote the occurrence and progression of HCC. To further elucidate the potential function and mechanism of TLK2 and avoid missing some DEGs with significant biological implications but having no significant differences in mRNA expression, the GSEA analysis of DEGs was carried out in HBV-related HCC patients. According to GSEA analysis, G2M checkpoint, E2F targets, and MYC targets v1 were highly upregulated gene sets, which are hub pathways of cell cycle regulation and involved in cell growth and proliferation (Liberzon et al., 2015). TLK2’s role in promoting ASF1A function assists in recovering from G2 arrest post DNA damage (Bruinsma et al., 2016), yet its overexpression can disrupt the G2/M checkpoint and lead to genomic instability (Kim et al., 2016a). The E2F targets pathway is a vital intracellular signaling pathway for cell cycle regulation, there is uncontrolled cell proliferation after E2F activation (Kent and Leone, 2019). High E2F expression facilitates the initiation and development of HCC and predicts unfavorable outcomes (Lv et al., 2017; Huang et al., 2019). MYC oncogene overexpression promotes tumorigenesis in various tumors, including liver cancer (Stine et al., 2015). Hepatocarcinogenesis is a multi-factor and complex process, certain intricate interplay of pathways may be involved. The above results provide directions for further research on the molecular mechanism of TLK2.

To our knowledge, this study first reported that TLK2 was highly expressed in HBV-related HCC with a poor prognosis. Also, we have some limitations that need to be noted. First, we use the data of public databases for statistical analysis, while databases are constantly updated and supplemented, the outcomes might be influenced. Second, our model was constructed specifically for HBV-related HCC patients and the generalizability of the model in HCC patients with other etiologies is unclear. Therefore, further validation of the prognostic value of TLK2 in large sample size, multicentered prospective studies is required. Finally, the research on the potential molecular mechanism of TLK2 in HCC is insufficient, and it lacks the verification of *in vivo* and *in vitro* experiments.

We confirm that TLK2 was highly expressed in HBV-related HCC and as an independent prognostic factor for OS and RFS. Furthermore, TLK2 is closely related to immune infiltrating cells and key molecules of signaling pathways and is involved in DNA replication, DNA repair, and cell cycle regulation, which may contribute to the onset and progression of HCC. Therefore, TLK2 could serve as a potential prognostic marker for HBV-related HCC.

## Data Availability

The original contributions presented in the study are included in the article/Supplementary Material, further inquiries can be directed to the corresponding author.
