# Supplementary figures and images for: Overexpression of tousled-like kinase 2 predicts poor prognosis in HBV-related hepatocellular carcinoma patients after radical resection
Source: Front Genet. 2024 Jan 26;14:1326737. doi: 10.3389/fgene.2023.1326737 (PMC10853388; doi:10.3389/fgene.2023.1326737)

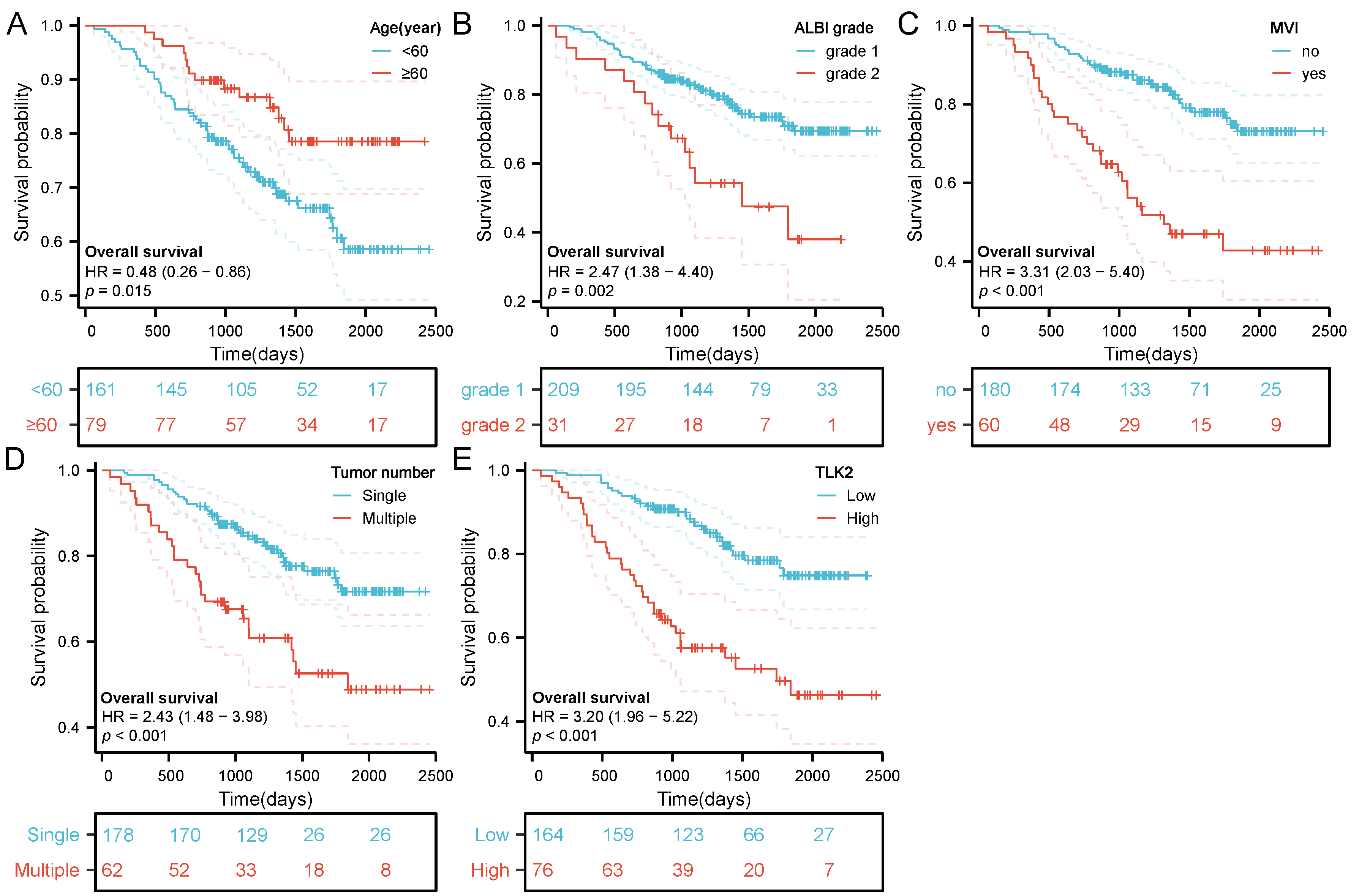

Supplement: Supplementary file 1 [file Image1.TIFF]

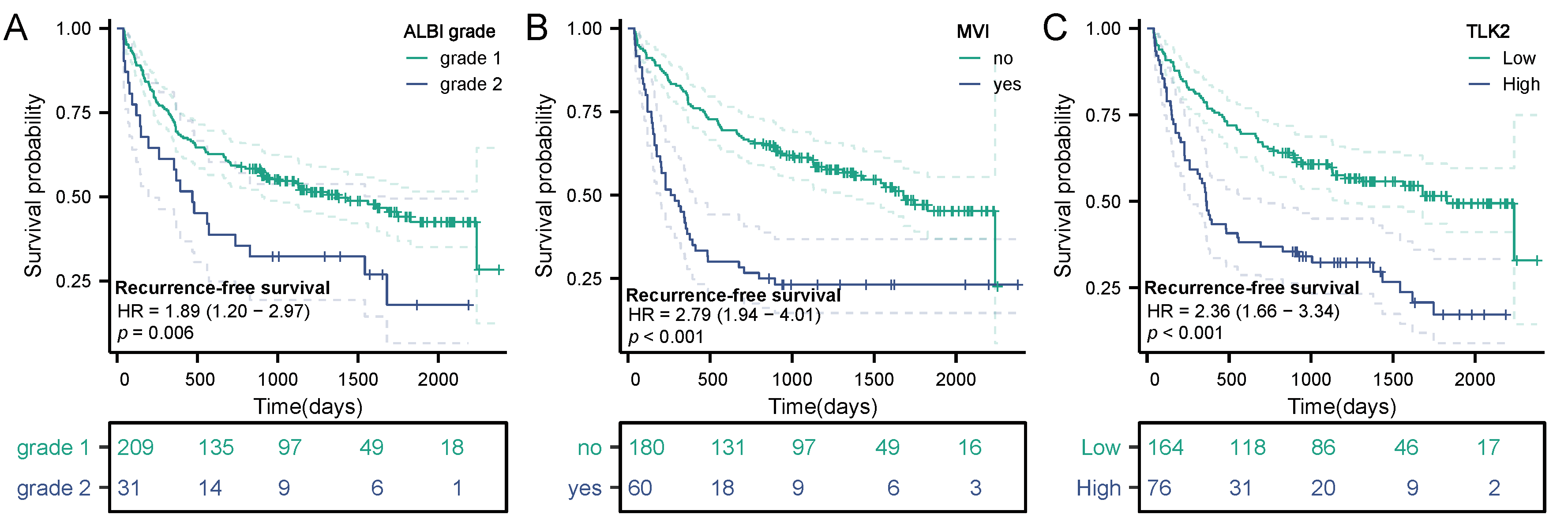

Supplement: Supplementary file 6 [file Image2.TIFF]
