# Supplementary material for: Overexpression of tousled-like kinase 2 predicts poor prognosis in HBV-related hepatocellular carcinoma patients after radical resection
Source: Front Genet. 2024 Jan 26;14:1326737. doi: 10.3389/fgene.2023.1326737 (PMC10853388; doi:10.3389/fgene.2023.1326737)
Supplement: Supplementary file 2 [file Table1.docx]

**Supplementary Tables S1.** Cox regression analysis for OS in the TCGA LIHC dataset.

| **Characteristics** | **Total(N)** | **Univariate analysis** | | **Multivariate analysis** | |
| --- | --- | --- | --- | --- | --- |
|  |  | **Hazard ratio (95% CI)** | ***p* value** | **Hazard ratio (95% CI)** | ***p* value** |
| **Gender**, female vs. male | 121/252 | 0.793 (0.557 - 1.130) | 0.200 |  |  |
| **Age**, <60 vs. ≥60 | 177/196 | 1.205 (0.850 - 1.708) | 0.295 |  |  |
| **Race** | 361 |  |  |  |  |
| Asian | 159 | Reference |  |  |  |
| Black or African American | 17 | 1.585 (0.675 - 3.725) | 0.290 |  |  |
| White | 185 | 1.323 (0.909 - 1.928) | 0.144 |  |  |
| **BMI**, ≤ 25 vs. > 25 | 177/159 | 0.798 (0.550 - 1.158) | 0.235 |  |  |
| **Residual tumor**, R0 vs. R1&R2 | 326/18 | 1.604 (0.812 - 3.169) | 0.174 |  |  |
| **AFP**, ≤ 400 vs. > 400 | 215/64 | 1.075 (0.658 - 1.759) | 0.772 |  |  |
| **Child-Pugh class**, A vs. B&C | 218/22 | 1.643 (0.811 - 3.330) | 0.168 |  |  |
| **Histologic grade** | 368 |  |  |  |  |
| G1 | 55 | Reference |  |  |  |
| G2 | 178 | 1.162 (0.686 - 1.969) | 0.576 |  |  |
| G3 | 123 | 1.185 (0.683 - 2.057) | 0.545 |  |  |
| G4 | 12 | 1.681 (0.621 - 4.549) | 0.307 |  |  |
| **Adjacent hepatic tissue inflammation** | 236 |  |  |  |  |
| none | 118 | Reference |  |  |  |
| mild | 101 | 1.204 (0.723 - 2.007) | 0.476 |  |  |
| severe | 17 | 1.144 (0.447 - 2.930) | 0.779 |  |  |
| **Vascular invasion**, no vs. yes | 208/109 | 1.344 (0.887 - 2.035) | 0.163 |  |  |
| **Pathologic T stage** | 370 |  |  |  |  |
| T1 | 183 | Reference |  | Reference |  |
| T2 | 94 | 1.431 (0.902 - 2.268) | 0.128 | 1.533 (0.839 - 2.801) | 0.165 |
| T3 | 80 | 2.674 (1.761 - 4.060) | < 0.001 | 2.635 (1.532 - 4.533) | **< 0.001** |
| T4 | 13 | 5.386 (2.690 - 10.784) | < 0.001 | 4.818 (1.774 - 13.083) | **0.002** |
| **Pathologic N stage**, N0 vs. N1 | 254/4 | 2.029 (0.497 - 8.281) | 0.324 |  |  |
| **Pathologic M stage**, M0 vs. M1 | 268/4 | 4.077 (1.281 - 12.973) | 0.017 | 0.775 (0.149 - 4.031) | 0.762 |
| **Tumor status**, tumor free vs. with tumor | 202/152 | 2.317 (1.590 - 3.376) | < 0.001 | 1.779 (1.107 - 2.858) | **0.017** |
| **TLK2**, low vs. high | 187/186 | 1.634 (1.152 - 2.317) | 0.006 | 1.626 (1.023 - 2.583) | **0.040** |

**Abbreviations:** BMI, body mass index; AFP, alpha-fetoprotein; TP53, tumor protein P53; TLK2, tousled-like kinase 2.
