# Supplementary material for: Overexpression of tousled-like kinase 2 predicts poor prognosis in HBV-related hepatocellular carcinoma patients after radical resection
Source: Front Genet. 2024 Jan 26;14:1326737. doi: 10.3389/fgene.2023.1326737 (PMC10853388; doi:10.3389/fgene.2023.1326737)
Supplement: Supplementary file 3 [file Table2.DOCX]

**Supplementary Tables 2.** Top ten hub proteins of TLK2’s interacting proteins obtained by five algorithms of the cytoHubba plugin in Cytoscape software

| **Catelogy** | **MCC** | **DMNC** | **EPC** | **Betweenness** | **Stress** |
| --- | --- | --- | --- | --- | --- |
| Top 10 hub proteins | **MCM2** | NCAPH | TLK1 | TLK1 | TLK1 |
|  | **MCM7** | MCM3 | ORC4 | ASF1A | ASF1A |
|  | ASF1B | ASF1B | ASF1A | ORC4 | ORC4 |
|  | ASF1A | **MCM7** | **MCM7** | RBM39 | RBM39 |
|  | ORC4 | **MCM2** | ASF1B | **GINS1** | **MCM7** |
|  | MCM3 | RAD9A | **MCM2** | PSMF1 | **MCM2** |
|  | NCAPH | EPB41L4A | TOP1 | TOP1 | TOP1 |
|  | **GINS1** | CEP76 | NCAPH | **MCM7** | PSMF1 |
|  | TLK1 | SMPD2 | MCM3 | **MCM2** | ASF1B |
|  | TOP1 | **GINS1** | **GINS1** | AMN1 | **GINS1** |

Abbreviations: MCC: Maximal cilque centrality; DMNC: density of maximum neighbourhood component; EPC: Edge percolated component.
