# Supplementary material for: Overexpression of tousled-like kinase 2 predicts poor prognosis in HBV-related hepatocellular carcinoma patients after radical resection
Source: Front Genet. 2024 Jan 26;14:1326737. doi: 10.3389/fgene.2023.1326737 (PMC10853388; doi:10.3389/fgene.2023.1326737)
Supplement: Supplementary file 4 [file Table3.DOCX]

**Supplementary Tables 3.** Top ten hub genes of DEGs obtained by five algorithms of the cytoHubba plugin in Cytoscape software

| **Catelogy** | **MCC** | **Radiality** | **Degree** | **EPC** | **Closeness** |
| --- | --- | --- | --- | --- | --- |
| Top 10 hub genes | **SLC22A7** | **SERPINA4** | **SLC22A7** | **SLC22A7** | **SLC22A7** |
|  | **CYP7A1** | **PON1** | **CYP7A1** | **CYP7A1** | **CYP7A1** |
|  | NR1I3 | SPP2 | **CYP4A11** | **CYP4A11** | **CYP4A11** |
|  | SLC51A | HGFAC | NR1I3 | NR1I3 | NR1I3 |
|  | **CYP4A11** | HPR | SLC51A | SLC51A | SLC51A |
|  | **PON1** | APOF | **PON1** | **PON1** | **PON1** |
|  | **SERPINA4** | **SLC22A7** | HPR | SLC22A1 | SLC22A1 |
|  | SPP2 | **CYP7A1** | APOF | AKR1D1 | AKR1D1 |
|  | HGFAC | **CYP4A11** | **SERPINA4** | **SERPINA4** | **SERPINA4** |
|  | HPR | MASP2 | SPP2 | HPR | CYP4F3 |

**Abbreviations:** MCC: Maximal cilque centrality; Degree: Node connect degree; EPC: Edge percolated component.
