# Supplementary material for: Overexpression of tousled-like kinase 2 predicts poor prognosis in HBV-related hepatocellular carcinoma patients after radical resection
Source: Front Genet. 2024 Jan 26;14:1326737. doi: 10.3389/fgene.2023.1326737 (PMC10853388; doi:10.3389/fgene.2023.1326737)
Supplement: Supplementary file 5 [file Table4.DOCX]

**Supplementary Tables 4.** GSEA analysis of significantly enriched Hallmark gene sets by using GSE121248.

| **Description** | **setSize** | **ES** | **NES** | ***p* value** | ***p*.adjust** | ***q* value** |
| --- | --- | --- | --- | --- | --- | --- |
| HALLMARK_G2M_CHECKPOINT | 185 | 0.7214223 | 2.916165 | 1e-10 | 6.25e-10 | 2.11e-10 |
| HALLMARK_E2F_TARGETS | 190 | 0.7134373 | 2.889374 | 1e-10 | 6.25e-10 | 2.11e-10 |
| HALLMARK_XENOBIOTIC_METABOLISM | 192 | -0.7599730 | -2.649805 | 1e-10 | 6.25e-10 | 2.11e-10 |
| HALLMARK_BILE_ACID_METABOLISM | 109 | -0.7679918 | -2.495694 | 1e-10 | 6.25e-10 | 2.11e-10 |
| HALLMARK_MYC_TARGETS_V1 | 184 | 0.6104725 | 2.471303 | 1e-10 | 6.25e-10 | 2.11e-10 |
| HALLMARK_COAGULATION | 136 | -0.7123905 | -2.390916 | 1e-10 | 6.25e-10 | 2.11e-10 |
| HALLMARK_FATTY_ACID_METABOLISM | 152 | -0.6454527 | -2.203620 | 1e-10 | 6.25e-10 | 2.11e-10 |
| HALLMARK_MITOTIC_SPINDLE | 198 | 0.5373981 | 2.181823 | 1e-10 | 6.25e-10 | 2.11e-10 |
| HALLMARK_INTERFERON_GAMMA_RESPONSE | 194 | -0.5533105 | -1.932071 | 8.93e-09 | 4.96e-08 | 1.67e-08 |
| HALLMARK_SPERMATOGENESIS | 132 | 0.5059745 | 1.961391 | 3.42e-07 | 1.71e-06 | 5.75e-07 |

**Abbreviations:** ES: enrichment score; NES, normalized enrichment score.
